# Vitamin D Deficiency and Its Association With Hypertension and Dyslipidemia in a Resource-Limited Cardiovascular Clinic Cohort in Yemen: A Cross-Sectional Analysis

**DOI:** 10.7759/cureus.93551

**Published:** 2025-09-30

**Authors:** Nabil Albaadani, Basheer Abdo, Mohammed Abdullah, Khaled H Alzanen, Ismaeel A Alshoaibi, Mohammed Almogahed

**Affiliations:** 1 Department of Internal Medicine, School of Medicine, Ibb University, Ibb, YEM

**Keywords:** 25-hydroxyvitamin d, cardiometabolic risk factors, dyslipidemia, hypertension, hypertriglyceridemia, lipid metabolism, vitamin d deficiency, yemen

## Abstract

Background: Vitamin D deficiency is a prevalent public health issue that disproportionately affects populations in resource-limited regions, even in the presence of abundant sunlight. Cultural practices and healthcare system limitations across the Middle East contribute to elevated rates of deficiency, which, in turn, worsen the burden of cardiometabolic diseases. This study aims to assess the prevalence of vitamin D deficiency and explore its association with hypertension and dyslipidemia in a clinical cohort from Yemen - a setting where diagnostic challenges complicate disease management.

Patients and methods: A retrospective cross-sectional study was implemented, enrolling 404 adult patients who attended a tertiary cardiovascular clinic at Ibb University, Ibb, Yemen, from January to December 2023. Serum 25-hydroxyvitamin D (25(OH)D) and fasting lipid parameters were measured. Vitamin D status was classified per Endocrine Society guidelines as deficient (<20 ng/mL), insufficient (20-29 ng/mL), or sufficient (≥30 ng/mL). Both multivariable linear and logistic regression analyses were utilized to determine independent predictors of vitamin D status, adjusting for demographic and clinical variables, including age, sex, hypertension, and lipid parameters.

Results: The study cohort had a mean age of 43.0 ± 16.0 years, with females comprising 52.5% (n = 212). Hypertension was present in 45.8% (n = 185) of participants. Vitamin D status classification revealed that 36.6% (n = 148) of the cohort were deficient, 31.2% (n = 126) insufficient, and 32.2% (n = 130) had sufficient vitamin D levels. Adjusted multivariate analyses indicated that hypertriglyceridemia (≥200 mg/dL) was associated with an 89% increased odds of vitamin D deficiency (adjusted odds ratio (aOR) = 1.89; 95% confidence interval (CI), 1.09-3.28; p = 0.024), while hypertension conferred a 61% increase in the odds of deficiency (aOR = 1.61; 95% CI, 1.04-2.50; p = 0.034). Elevated high-density lipoprotein cholesterol (HDL-C) (≥60 mg/dL) exhibited a protective effect against deficiency (aOR = 0.41; 95% CI, 0.20-0.84; p = 0.015). Furthermore, each 10-year increment in age was associated with a 22% higher likelihood of vitamin D deficiency (aOR = 1.22; 95% CI, 1.00-1.48; p = 0.015).

Conclusion: In this resource-constrained Yemeni clinical cohort, vitamin D deficiency is independently associated with hypertriglyceridemia, low HDL-C, and hypertension. These findings underscore the importance of targeted vitamin D screening and integrated cardiometabolic risk management strategies within similar vulnerable populations, and highlight the need for further longitudinal studies to explore causality.

## Introduction

Vitamin D, a secosteroid hormone, is primarily synthesized in the skin through exposure to ultraviolet B (UVB) radiation. It plays a vital role in maintaining calcium homeostasis and supporting bone health [1]. Beyond these traditional functions, vitamin D is also involved in modulating cardiovascular health, immune responses, and glucose metabolism [2].

Globally, vitamin D deficiency, defined by serum 25-hydroxyvitamin D (25(OH)D) levels below 20 ng/mL, affects approximately one billion individuals [3]. Interestingly, populations in the Middle East exhibit a high prevalence of deficiency despite abundant sunlight exposure [3,4]. This paradox can be explained by factors such as cultural clothing practices that limit skin exposure, increased melanin pigmentation that reduces UVB penetration, and inadequate dietary intake [5]. Vitamin D deficiency has been linked to an increased risk of hypertension and dyslipidemia, thereby contributing to the burden of cardiometabolic diseases [5,6].

Vitamin D influences lipid metabolism, endothelial function, and insulin sensitivity - mechanisms that are highly relevant to cardiometabolic health [7-9]. However, evidence investigating the relationship between vitamin D deficiency and cardiovascular risk factors, particularly lipid subfractions and hypertension, remains limited in the Yemeni population and other resource-limited settings [10,11].

This study aims to determine the prevalence of vitamin D deficiency and to evaluate its independent associations with lipid abnormalities and hypertension within a resource-constrained clinical setting in Yemen. We hypothesize that vitamin D deficiency will be highly prevalent in this cohort and will be independently associated with increased odds of hypertension and adverse lipid profiles. The findings are intended to inform culturally and regionally tailored screening and intervention strategies to reduce cardiometabolic risk in this high-risk population.

## Materials and methods

Study design and setting

This retrospective cross-sectional study was conducted at a single-center private laboratory affiliated with Ibb University, Ibb, Yemen, over the period from January to December 2023. The study protocol received ethical approval from the Institutional Ethics Committee of Ibb University (Approval ID: IBBUNI.AC.YEM.2025.12; approved on January 2, 2025). Given the retrospective design and utilization of de-identified data, the requirement for individual informed consent was waived, in accordance with institutional policies and the principles outlined in the Declaration of Helsinki.

Study population

The study cohort comprised 404 adults aged 18 years and older who underwent serum 25(OH)D testing within the study timeframe and satisfied predefined eligibility criteria. Exclusion criteria included pregnancy, lactation, documented hepatic or renal insufficiency, current vitamin D supplementation exceeding 400 IU/day, and diagnosed malabsorption syndromes. Consecutive sampling was employed to reduce the risk of selection bias.

Sample size calculation

Using G Power 3.1 (Heinrich-Heine-Universität Düsseldorf, Düsseldorf, Germany), the sample size was calculated to detect a clinically significant association between vitamin D deficiency and hypertension, with 90% power at a 5% significance level. Based on regional data from Palestine [12], with a vitamin D deficiency prevalence of 78% and a hypertension prevalence of 20.8%, an allocation ratio of approximately 3.5:1 was assumed. To detect an odds ratio (OR) of 1.6, the minimum required sample size was 346 participants [9]. Our final sample of 404 exceeded this, ensuring sufficient power to evaluate the associations.

Data collection

Demographic, clinical, and biochemical data were extracted retrospectively from electronic health records and laboratory databases. Information regarding smoking status, Qat chewing habits, and medical history was obtained from structured fields within the records. Blood pressure (BP) was measured using calibrated sphygmomanometers per standardized protocols; hypertension was defined as systolic BP ≥140 mmHg, diastolic BP ≥90 mmHg, or antihypertensive use. Anthropometrics (height, weight, and waist circumference) were obtained following WHO protocols, where available [13].

Laboratory analysis

Fasting venous blood collection occurred between 07:00 and 09:00 after a 12-hour fast, as per local laboratory protocol to ensure standardization and minimize postprandial lipemia. Serum 25(OH)D concentrations were quantified by chemiluminescent immunoassay using the LIAISON® XL Analyzer (DiaSorin Inc., Stillwater, MN, USA), achieving an inter-assay coefficient of variation of 5.2%, indicative of acceptable assay precision. All laboratory assays were performed following strict internal quality control protocols, including daily calibration and the use of internal quality control materials. Lipid profiles - including total cholesterol, triglycerides, high-density lipoprotein cholesterol (HDL-C), and low-density lipoprotein cholesterol (LDL-C) - were measured enzymatically on the Roche Cobas® 8000 platform (Roche Diagnostics GmbH, Mannheim, Germany). Lipid abnormalities were classified in accordance with National Cholesterol Education Program Adult Treatment Panel III (NCEP ATP III) criteria [14]. Vitamin D status was defined according to the Endocrine Society Clinical Practice Guidelines: deficiency (<20 ng/mL), insufficiency (20-29 ng/mL), or sufficiency (≥30 ng/mL) [15].

Handling of missing data

Cases missing essential data for vitamin D or lipid profiles were excluded from analyses. Fewer than 20 cases (<5% of the total sample) were excluded due to missing essential vitamin D or lipid data. A comparison of basic demographics between included and excluded cases showed no significant differences, making systematic bias unlikely. Imputation methods were not utilized due to the retrospective design.

Statistical analysis

All analyses were performed using IBM SPSS Statistics for Windows, Version 27 (Released 2019; IBM Corp., Armonk, NY, USA). Continuous variables were presented as mean ± standard deviation, and compared across vitamin D categories using one-way analysis of variance (ANOVA), followed by Tukey’s post hoc test for pairwise comparisons. Categorical variables were summarized as frequencies and percentages, and group comparisons were conducted via Pearson’s Chi-square test or Fisher’s exact test, as appropriate.

Univariate linear regression analyses identified potential predictors of serum 25(OH)D levels; variables with p < 0.10 were entered into multivariable linear regression models. Logistic regression was used to determine factors associated with vitamin D deficiency (serum 25(OH)D < 20 ng/mL), with ORs and 95% confidence intervals (CIs) reported. Model diagnostics included assessment of residual normality using the Shapiro-Wilk test, evaluation of multicollinearity via variance inflation factors (VIF < 5), and goodness-of-fit testing with the Hosmer-Lemeshow test. Post hoc power analysis confirmed that the study had over 90% power to detect differences in primary outcomes. A p-value less than 0.005 was considered statistically significant.

## Results

The study included 404 adult patients with a mean age of 43.0 ± 16.0 years; females comprised 212 (52.5%) of the cohort (Table 1). Hypertension was present in 185 individuals (45.8%), with 102 (25.2%) identifying as current smokers and 88 (21.8%) reporting habitual Qat chewing. Vitamin D status classification revealed that 36.6% (n = 148) of the cohort were deficient (<20 ng/mL), 31.2% (n = 126) insufficient (20-29 ng/mL), and 32.2% (n = 130) had sufficient vitamin D levels (≥30 ng/mL) (Table 1). Notable differences were observed among these groups: the deficient cohort was significantly older (mean age 45.2 ± 15.8 years) compared to those with insufficient and sufficient vitamin D levels (42.1 ± 16.2 and 41.5 ± 15.9 years, respectively; p = 0.042). The prevalence of hypertension was also highest among the deficient group (52.7%) relative to the insufficient (44.4%) and sufficient groups (39.2%) (p = 0.048). No significant group differences were found with respect to sex, smoking status, or Qat chewing.

**Table 1:** Demographic and Clinical Characteristics According to Vitamin D Status Categories *p-values correspond to overall group comparisons using ANOVA for continuous variables and Chi-square tests for categorical variables. †Lipid profile categories are defined per National Cholesterol Education Program Adult Treatment Panel III (NCEP ATP III) criteria [10]. SD, standard deviation; HDL, high-density lipoprotein cholesterol; LDL, low-density lipoprotein cholesterol; ANOVA, analysis of variance

Characteristic	Overall (N = 404)	Deficient (<20 ng/mL) (n = 148)	Insufficient (20-29 ng/mL) (n = 126)	Sufficient (≥30 ng/mL) (n = 130)	p-value
Age (years), mean ± SD	43.0 ± 16.0	45.2 ± 15.8	42.1 ± 16.2	41.5 ± 15.9	0.042*
Sex, n (%)					0.245
Male	192 (47.5)	65 (43.9)	60 (47.6)	67 (51.5)	-
Female	212 (52.5)	83 (56.1)	66 (52.4)	63 (48.5)	-
Hypertension, n (%)	185 (45.8)	78 (52.7)	56 (44.4)	51 (39.2)	0.048*
Smoking, n (%)	102 (25.2)	40 (27.0)	32 (25.4)	30 (23.1)	0.718
Qat Chewing, n (%)	88 (21.8)	35 (23.6)	28 (22.2)	25 (19.2)	0.73
Lipid Profile, n (%)†					
Total Cholesterol					0.21
<200 mg/dL (Desirable)	220 (54.5)	78 (52.7)	68 (54.0)	74 (56.9)	-
200-239 mg/dL (Borderline)	120 (29.7)	44 (29.7)	38 (30.2)	38 (29.2)	-
≥240 mg/dL (High)	64 (15.8)	26 (17.6)	20 (15.8)	18 (13.9)	-
Triglycerides					0.009*
<150 mg/dL (Normal)	152 (37.6)	42 (28.4)	52 (41.3)	58 (44.6)	-
150-199 mg/dL (Borderline)	126 (31.2)	54 (36.5)	38 (30.2)	34 (26.2)	-
≥200 mg/dL (High)	126 (31.2)	52 (35.1)	36 (28.6)	38 (29.2)	-
HDL Cholesterol					0.005*
<40 mg/dL (Low)	168 (41.6)	76 (51.4)	50 (39.7)	42 (32.3)	-
40-59 mg/dL (Normal)	172 (42.6)	56 (37.8)	56 (44.4)	60 (46.2)	-
≥60 mg/dL (High)	64 (15.8)	16 (10.8)	20 (15.9)	28 (21.5)	-
LDL Cholesterol					0.037*
<100 mg/dL (Optimal)	280 (69.3)	96 (64.9)	96 (76.2)	88 (67.7)	-
100-129 mg/dL (Near Optimal)	72 (17.8)	28 (18.9)	18 (14.3)	26 (20.0)	-
130-159 mg/dL (Borderline)	36 (8.9)	16 (10.8)	8 (6.3)	12 (9.2)	-
≥160 mg/dL (High)	16 (4.0)	8 (5.4)	4 (3.2)	4 (3.1)	-

Analysis of serum lipid profiles revealed significant differences among the vitamin D status groups (Table 1). Elevated triglyceride levels (≥200 mg/dL) were more prevalent in the vitamin D-deficient group, with 52 of 148 patients (35.1%) affected, compared to 36 of 126 (28.6%) in the insufficient group and 38 of 130 (29.2%) in the sufficient group (p = 0.009). Low HDL-C (<40 mg/dL) was most frequent among those deficient in vitamin D, observed in 76 of 148 patients (51.4%), versus 50 of 126 (39.7%) and 42 of 130 (32.3%) in the insufficient and sufficient groups, respectively (p = 0.005). Borderline and high LDL-C (≥130 mg/dL) levels were also disproportionately higher in the deficient group, with 24 of 148 patients (16.2%) affected, compared to 12 of 126 (9.5%) and 16 of 130 (12.3%) in the insufficient and sufficient groups (p = 0.037). No significant differences were observed between groups for total cholesterol categories.

Univariate linear regression identified significant inverse relationships between serum 25(OH)D levels and age (β = -0.11 per year; 95% CI, -0.18 to -0.04; p = 0.002), hypertension (β = -2.67; 95% CI, -4.71 to -0.63; p = 0.010), and triglycerides ≥200 mg/dL (β = -3.78; 95% CI, -6.12 to -1.44; p = 0.002) (Table 2). Conversely, HDL-C ≥60 mg/dL correlated positively with vitamin D concentrations (β = 4.25; 95% CI, 1.32 to 7.18; p = 0.005). Other variables, including sex, total cholesterol, and LDL cholesterol, were not significant predictors in the univariate model.

**Table 2:** Univariate Linear Regression Analysis of Predictors for Serum 25-Hydroxyvitamin D Levels (ng/mL) *Statistically significant at p < 0.05. β, regression coefficient; CI, confidence interval; HDL, high-density lipoprotein; LDL, low-density lipoprotein

Predictor	Category (Reference)	β Coefficient (95% CI)	p-value
Age (per year)	-	-0.11 (-0.18, -0.04)	0.002*
Sex	Male (Reference)	-	0.435
	Female	0.82 (-1.24, 2.88)	
Hypertension	No (Reference)	-	0.010*
	Yes	-2.67 (-4.71, -0.63)	
Total Cholesterol	<200 mg/dL (Reference)	-	0.124
	200-239 mg/dL	-1.85 (-4.21, 0.51)	
	≥240 mg/dL	-3.12 (-7.01, 0.77)	
Triglycerides	<150 mg/dL (Reference)	-	0.002*
	150-199 mg/dL	-2.31 (-4.62, 0.00)	0.05
	≥200 mg/dL	-3.78 (-6.12, -1.44)	
HDL Cholesterol	<40 mg/dL (Reference)	-	0.005*
	40-59 mg/dL	1.92 (-0.14, 3.98)	0.068
	≥60 mg/dL	4.25 (1.32, 7.18)	
LDL Cholesterol	<100 mg/dL (Reference)	-	0.188
	100-129 mg/dL	-1.05 (-3.68, 1.58)	0.433
	≥130 mg/dL	-2.47 (-6.15, 1.21)	

As presented in Table 3, multivariable logistic regression modeling revealed a significant positive association between advancing age and vitamin D deficiency, with each incremental decade conferring a 22% elevation in adjusted odds (aOR = 1.22; 95% CI, 1.00-1.48; p = 0.015). Hypertension independently increased the likelihood of deficiency by 61% (aOR = 1.61; 95% CI, 1.04-2.50; p = 0.034), while hypertriglyceridemia (≥200 mg/dL) was associated with a near doubling of risk (aOR = 1.89; 95% CI, 1.09-3.28; p = 0.024). Conversely, elevated HDL-C (≥60 mg/dL) demonstrated a protective effect, reducing deficiency odds by 59% (aOR = 0.41; 95% CI, 0.20-0.84; p = 0.015). Sex and total cholesterol levels did not reach statistical significance in the final model. The predictive accuracy of the regression model was robust (area under the curve (AUC) = 0.74; 95% CI, 0.69-0.79), with appropriate calibration (Hosmer-Lemeshow p = 0.55).

**Table 3:** Determinants of Vitamin D Deficiency (Serum 25-Hydroxyvitamin D < 20 ng/mL) Identified by Univariable and Multivariable Logistic Regression Analysis *Statistically significant at p < 0.05. Crude ORs and 95% CIs were estimated with univariable logistic regression. Fully adjusted ORs and 95% CIs were obtained from a multivariable logistic regression model that simultaneously included all covariates listed. Vitamin D deficiency was operationalized as a serum 25-hydroxyvitamin D concentration below 20 ng/mL. Reference categories for each categorical predictor are explicitly indicated. The predictive accuracy of the regression model was robust (AUC = 0.74; 95% CI, 0.69-0.79). OR, odds ratio; CI, confidence interval; HDL, high-density lipoprotein; AUC, area under the curve

Predictor	Category (Reference)	Unadjusted OR (95% CI)	p-value (Unadjusted)	Adjusted OR (95% CI)	p-value (Adjusted)
Age (per 10-year increase)	Continuous	1.18 (0.98-1.42)	0.078	1.22 (1.00-1.48)	0.015*
Sex	Male	Reference	-	Reference	-
	Female	1.10 (0.83-1.46)	0.525	1.05 (0.78-1.42)	0.735
Hypertension	No	Reference	-	Reference	-
	Yes	1.49 (1.01-2.20)	0.045*	1.61 (1.04-2.50)	0.034*
Triglycerides	<150 mg/dL	Reference	-	Reference	-
	150-199 mg/dL	1.25 (0.76-2.06)	0.381	1.32 (0.78-2.23)	0.298
	≥200 mg/dL	1.75 (1.07-2.87)	0.027*	1.89 (1.09-3.28)	0.024*
HDL Cholesterol	<40 mg/dL	Reference	-	Reference	-
	40-59 mg/dL	0.78 (0.48-1.26)	0.309	0.72 (0.44-1.18)	0.195
	≥60 mg/dL	0.45 (0.23-0.89)	0.022*	0.41 (0.20-0.84)	0.015*
Total Cholesterol	<200 mg/dL	Reference	-	Reference	-
	200-239 mg/dL	1.05 (0.64-1.74)	0.845	1.12 (0.67-1.87)	0.667
	≥240 mg/dL	1.18 (0.60-2.31)	0.632	1.28 (0.63-2.60)	0.491

## Discussion

This study identified significant independent correlations between dyslipidemia, hypertension, and vitamin D deficiency within a clinical population from a resource-limited Middle Eastern region. The observed vitamin D deficiency prevalence of 36.6% is consistent with reports from Gulf Cooperation Council countries and Yemen [10,11], where deficiency rates frequently exceed 30%, despite abundant sunlight [4,6,16]. Cultural practices limiting skin exposure, increased skin pigmentation, dietary insufficiencies, and lifestyle factors, such as reduced outdoor activity under extreme temperatures, contribute to this paradoxical prevalence [5,17]. A comparable investigation by Madkhali et al. reported a 67.3% period prevalence of vitamin D deficiency within a large Saudi Arabian cohort, further underscoring this persistent and widespread public health concern in the region [4]. These findings highlight the critical importance of integrating vitamin D status assessment into national cardiometabolic risk screening initiatives in resource-constrained Middle Eastern settings like Yemen, to facilitate timely detection and targeted intervention.

In this study, even after adjusting for age, sex, and lipid profiles, individuals with hypertriglyceridemia and hypertension were independently more likely to experience vitamin D deficiency, with aORs of 1.89 and 1.61, respectively. Notably, having HDL-C levels of 60 mg/dL or higher appeared to confer a protective effect, reflected by an aOR of 0.41. These strong associations, particularly for hypertriglyceridemia, exceed those reported in some Western populations [18,19]. While these findings are compelling, it is important to recognize that the cross-sectional design of this study limits causal inference. The observed relationships may be influenced by unmeasured confounders, such as physical activity, dietary habits, socioeconomic status, or sun exposure, which were not fully accounted for, and could impact both vitamin D levels and cardiometabolic outcomes.

The relatively stronger associations observed in our population may partly be explained by genetic differences, such as vitamin D receptor (VDR) gene polymorphisms, which are prevalent among Arab populations. These polymorphisms could influence how vitamin D interacts with cardiometabolic pathways, though further genetic and functional studies are needed to clarify these mechanisms. Existing mechanistic research supports the role of vitamin D in lipid metabolism and vascular regulation. For example, vitamin D has been shown to suppress hepatic triglyceride synthesis by downregulating sterol regulatory element-binding protein-1c (SREBP-1c), and to promote triglyceride clearance through upregulation of lipoprotein lipase activity [20]. Additionally, vitamin D influences the renin-angiotensin-aldosterone system, which plays a key role in vascular tone and BP regulation [19]. These findings underscore the potential of vitamin D deficiency as both a biomarker and a modifiable risk factor within the broader context of cardiometabolic health. However, while mechanistic and observational data suggest a beneficial role for vitamin D, randomized controlled trials are necessary to establish whether vitamin D supplementation can effectively improve lipid profiles and BP regulation. Future research should focus on well-designed interventional studies that consider population-specific genetic factors, appropriate dosing, and relevant clinical outcomes.

The inverse relationship between serum vitamin D levels and triglyceride concentrations observed in our study aligns with findings from the Framingham Offspring Study and various other international cohorts [21-23]. Similarly, the positive association between vitamin D status and HDL-C supports evidence from populations in both the Middle East and Europe [4,17,21,24]. These collective observations highlight the clinical importance of assessing vitamin D levels in patients with dyslipidemia and hypertension, especially in regions with a high prevalence of deficiency. Such assessments could improve cardiovascular risk stratification and inform targeted supplementation strategies.

In this study, increasing chronological age was significantly associated with vitamin D deficiency, corroborating findings from previous reports that attribute this relationship to a diminished capacity for cutaneous vitamin D synthesis and alterations in vitamin D metabolism among older adults globally, including populations in the Middle East [4,6,25]. Although women exhibited a higher prevalence of deficiency, this difference did not reach statistical significance after adjustment in our analysis. Similarly, smoking status was not significantly associated with vitamin D deficiency, contrasting with some previous research [26,27]. These differences may be due to population-specific factors, such as variations in smoking intensity or unmeasured confounders. Within the regional context, socio-cultural practices - such as conservative clothing styles, reduced sun exposure, and habitual use of sun-protective measures among women - likely contribute to these observed patterns [6,28]. The complex interplay between demographic factors and metabolic dysfunction highlights their potential role in increasing susceptibility to cardiovascular morbidity in these populations. Importantly, the cross-sectional design of our study limits our ability to infer causality. The associations we observed may be explained by reverse causation, whereby underlying cardiometabolic conditions contribute to increased sedentary behavior and reduced outdoor activity, ultimately leading to lower vitamin D levels.

Several important factors influencing vitamin D status were not evaluated in this study, including dietary intake of vitamin D, physical activity, supplementation, BMI, medications, and comorbid conditions. The absence of these variables introduces potential residual confounding, with BMI being particularly significant given its strong association with both lower vitamin D levels and adverse cardiometabolic profiles [22]. Obesity, for example, can reduce circulating vitamin D by sequestering it in adipose tissue, while inadequate dietary intake limits substrate availability for synthesis [29]. Additionally, commonly prescribed medications, such as statins and antihypertensives, may influence vitamin D metabolism, though the mechanisms remain incompletely understood [30].

Environmental and cultural factors also play a crucial role in vitamin D synthesis. Variations in sunlight exposure due to seasonal changes, geographic location, and traditional clothing - especially relevant in the Yemeni context - can significantly diminish cutaneous vitamin D production [31]. We did not adjust for seasonal variation, which could have introduced unmeasured variability into our findings. Given that skin synthesis of vitamin D depends on adequate UVB exposure, factors like limited outdoor activity and skin coverage further increase deficiency risk [5,10,31]. Future research should incorporate these behavioral, environmental, and clinical variables to better understand their combined impact on vitamin D status and cardiometabolic health in similar populations.

The connection between khat chewing and vitamin D status remains poorly understood, with limited direct evidence in the literature. Although our study did not find a significant association, several plausible mechanisms suggest potential indirect links. Khat use is known to suppress appetite and reduce nutritional intake, which could lead to deficiencies in fat-soluble vitamins like vitamin D [32,33]. Additionally, compounds in khat, such as tannins and phenolics, may impair nutrient absorption by forming insoluble complexes, potentially affecting vitamin D bioavailability [34]. Khat-related gastrointestinal mucosal damage and higher rates of *Helicobacter pylori* infection might further compromise nutrient absorption [34]. Behavioral factors, such as reduced sun exposure due to social habits, could also influence endogenous vitamin D synthesis, although this has not been empirically examined [34,35]. Despite these theoretical considerations, our findings did not support a significant relationship between khat chewing and vitamin D deficiency, suggesting that khat may not be a major determinant in our cohort. Nevertheless, further research is warranted to clarify potential mechanisms and clinical relevance.

Similarly, while prior studies have reported an inverse relationship between tobacco use and serum 25(OH)D levels [26,27], our analysis did not reveal a significant association between smoking status and vitamin D deficiency. This suggests that, within our population, smoking may not play a primary role in determining vitamin D status, although additional research is needed to explore underlying factors and mechanisms.

Study limitations

This study has several methodological limitations. The retrospective, cross-sectional design restricts our ability to draw conclusions about causality or to establish temporal relationships between vitamin D deficiency and other cardiometabolic risk factors. Additionally, there is potential for selection bias, as our cohort was recruited from a single tertiary care center. While the demographic characteristics of this population broadly align with national health survey data, this may limit the generalizability of our findings to the wider Yemeni population. The lack of data on key clinical and lifestyle variables - most notably BMI, dietary habits, and medication use - also introduces the possibility of residual confounding.

Although vitamin D and lipid levels were measured using standardized assays, variability between assays and calibration differences across laboratories may influence the precision of the absolute serum values. Our sample size limited the scope of subgroup analyses, particularly for less common, severe dyslipidemia phenotypes. Furthermore, we did not account for seasonal variations in vitamin D levels, which could impact our findings. Importantly, genetic factors that influence vitamin D metabolism - such as polymorphisms in the VDR and group-specific component (GC) genes - were not evaluated, representing a key area for future research.

Building on these findings, integrating vitamin D screening into existing hypertension and dyslipidemia management programs - especially in resource-limited settings - could improve early detection of deficiency using cost-effective, accessible assays. To validate and refine such strategies, future studies should focus on longitudinal designs to clarify causal relationships, and randomized controlled trials to assess the clinical benefits and cost-effectiveness of combined vitamin D supplementation and lipid-lowering therapies. Additionally, culturally appropriate public health initiatives promoting safe sunlight exposure - balancing the benefits against skin cancer risks - should be incorporated into comprehensive efforts to reduce vitamin D deficiency in these populations. Investigating the role of genetic polymorphisms affecting vitamin D metabolism within Middle Eastern populations will also be crucial for advancing personalized prevention and treatment strategies.

## Conclusions

This study demonstrates significant independent associations between hypertriglyceridemia, low HDL-C levels, and hypertension, with vitamin D deficiency in an adult clinical cohort from Yemen. Although the cross-sectional design limits causal interpretation, these findings highlight the potential benefit of targeted vitamin D screening in individuals with dyslipidemia and hypertension, especially in resource-limited settings.

Future research should focus on longitudinal studies to elucidate causal relationships, and interventional trials to assess the impact of vitamin D supplementation on cardiometabolic health. Additionally, exploring genetic polymorphisms influencing vitamin D metabolism within Middle Eastern populations may inform personalized approaches. Addressing vitamin D deficiency through integrated public health strategies could reduce the burden of cardiometabolic diseases in this vulnerable region.
